# Outpatient Physical Therapists Should be Competent in Care of Older Adults: A Total Population Register-Based Study

**DOI:** 10.1093/geroni/igaa057.560

**Published:** 2020-12-16

**Authors:** Solveig Arnadottir

**Affiliations:** University of Iceland, Reykjavik, Iceland

## Abstract

In Iceland, outpatient physical therapy (OPT) is traditionally not focused on older clients. Yet, the Icelandic population is aging as other populations in the world, and national policies endorse aging in place. The objective of this study was to explore 17 years of demographic information on OPT clients and to identify if this information reflects the total population aging. The research was built on 17 years (1999-2015) of complete data from: the Icelandic Health Insurances register with information on the total population of OPT clients (N=172071), and the Statistics Iceland register with demographic information on the total general population. The results revealed that in 1999, older adults comprised 18.3% of all OPT clients, and in 2015 it had increased to 23.5% Therefore, OPTs were 23% more likely to treat an older adult in 2015, compared to 1999 (Risk Ratio [RR] 1.23; 95% Confidence Interval [CI] 1.19-1.27). In the same time period older people became 15% more prevalent in the general population (RR 1.15; 95%CI 1.10-1.21). Linear modelling revealed a yearly 3.45% (95%CI 3.05-3.85) increase from 1999 to 2015 in the overall proportion of older OPT clients. This yearly trend, however, varied depending on age group and sex with the highest yearly increase in the ≥ 85 years old men (9.1%; 95%CI 7.90-10.35). This case of Iceland presents 17 years of continuous growth in older adults seeking OPT service. These findings reinforce an urgent need to enhance the geriatric competence of OPTs, who in their clinical practice frequently encounter older adults.

